# Switching to the Rubber Hand

**DOI:** 10.3389/fpsyg.2017.02172

**Published:** 2017-12-12

**Authors:** Su-Ling Yeh, Timothy Joseph Lane, An-Yi Chang, Sung-En Chien

**Affiliations:** ^1^Department of Psychology, National Taiwan University, Taipei, Taiwan; ^2^Graduate Institute of Brain and Mind Sciences, National Taiwan University, Taipei, Taiwan; ^3^Neurobiology and Cognitive Science Center, National Taiwan University, Taipei, Taiwan; ^4^Graduate Institute of Humanities in Medicine, Taipei Medical University, Taipei, Taiwan; ^5^Research Center of Brain and Consciousness, Taipei Medical University, Taipei, Taiwan; ^6^Shuang Ho Hospital, Taipei Medical University, New Taipei City, Taiwan; ^7^Institute of European and American Studies, Academia Sinica, Taipei, Taiwan; ^8^Research Center for Mind, Brain, and Learning, National Chengchi University, Taipei, Taiwan

**Keywords:** rubber hand illusion, task switch, attention control, executive functions, body ownership

## Abstract

Inducing the rubber hand illusion (RHI) requires that participants look at an imitation hand while it is stroked in synchrony with their occluded biological hand. Previous explanations of the RHI have emphasized multisensory integration, and excluded higher cognitive functions. We investigated the relationship between the RHI and higher cognitive functions by experimentally testing task switch (as measured by switch cost) and mind wandering (as measured by SART score); we also included a questionnaire for attentional control that comprises two subscales, attention-shift and attention-focus. To assess experience of RHI, the Botvinick and Cohen (1998) questionnaire was used and illusion onset time was recorded. Our results indicate that rapidity of onset reliably indicates illusion strength. Regression analysis revealed that participants evincing less switch cost and higher attention-shift scores had faster RHI onset times, and that those with higher attention-shift scores experienced the RHI more vividly. These results suggest that the multi-sensory hypothesis is not sufficient to explain the illusion: higher cognitive functions should be taken into account when explaining variation in the experience of ownership for the rubber hand.

## Introduction

Plasticity of bodily experience, once a subject suited only to fictional characterization (Melzack, 1992) and philosophical speculation (de Vignemont, 2011a), has become a focus of intense experimental research. Tastevin (1937) laid the groundwork for this research with an observation concerning “visual capture”: the simple act of seeing a plastic finger that protrudes from a piece of cloth is sufficient to cause one to feel illusory sensations on one's concealed biological hand. This illusion seems to be caused by visual dominance over other sensory modalities. Botvinick and Cohen (1998) expanded upon Tastevin's finding by developing an experimental paradigm in which a participant's occluded biological hand is stroked in synchrony with a visible rubber hand. This induction procedure evokes what has been dubbed the “rubber hand illusion” (RHI)—among other things, tactile sensations seem to occur on the rubber hand and the rubber hand seems to belong to self (Botvinick, 2004).

The most commonly invoked explanation for the RHI, the Multi-Sensory Hypothesis (MSH) emphasizes multisensory integration among visual, somatosensory, proprioceptive, and other signals that operate within a body-centered coordinate system (Makin et al., 2008; Grivaz et al., 2017). Although the precise roles played by sensory signals like proprioception (Rohde et al., 2011; Abdulkarim and Ehrsson, 2016) and by peripersonal space (Makin et al., 2008) are contested, that the body's sensory signals are referenced to the body's position is widely accepted (e.g., Durgin et al., 2007). In other words, sensory information from both the body and the space adjacent to the body are integrated in such a way as to distinguish between what belongs to self and what belongs to the external world. Indeed, parts of that which is external to the body, the peripersonal, can be experienced as belonging to self. It is in virtue of this ongoing, dynamic process that body parts are felt to be owned by or belong to the self. Indeed, Ehrsson (2012, p. 788) avers that MSH is *sufficient* to explain body ownership, and that MSH has the added beauty of being parsimonious because it “does not require the inclusion of higher cognitive functions.”

Despite acknowledging that multisensory processing might be the main cause of the RHI, Tsakiris disputes the sufficiency claim (Tsakiris, 2010, 2011, 2017): First, he observes that visual form and anatomical congruency matter—i.e., the shape of a rubber hand must resemble an actual hand for it to be experienced as belonging to self (Armel and Ramachandran, 2003). Second, posture and spatial boundaries matter too: if the postures of rubber and biological hands are incongruent (Constantini and Haggard, 2007) or the distance between the two exceeds 30 cm (Lloyd, 2007), the illusion either disappears or is diluted. In other words, a pre-existing, normative model of the body constrains the RHI. Third, he adduces evidence to show that the “filtering” mechanism whereby an object is “tested” to see whether it coheres with the pre-existing model is independent of multisensory processing (e.g., Tsakiris et al., 2008). And, fourth, exteroceptive signals like vision and touch, must be integrated with interoceptive signals like those that emanate from the cardiovascular system (Tsakiris, 2011, 2017; Moseley et al., 2012). This integral relationship between exteroceptive and interoceptive signals is evinced in many other respects as well, including discovery that histamine reactivity increases in the biological hand, correlating with disownership experiences (Barnsley et al., 2011).

It seems clear that multi-sensory processes alone are not sufficient to explain the RHI, and that a normative body model as well as interoceptive processing need also be taken into account. Of course achieving a more complete explanation will require greater nuance as, for example, simple distance probably does not constrain in the way described above; a more complex model that limns the interplay of spatial and temporal parameters is needed (Zopf et al., 2010; Constantini et al., 2016). But these additions do contribute to explanatory adequacy, while at the same time being broadly consistent with Ehrsson's desideratum that the explanation be parsimonious, because like MSH neither of these additions involve higher cognitive functions.

Adequate scientific explanations, however, should do more than merely explain what happens; they should also explain why, under like circumstances, there is variability, even the failure to occur (Lipton, 2004). Evidence from multiple investigations—evidence that is consistent with our findings—shows that when standardized induction procedures for the RHI are employed, variability is the norm (e.g., Botvinick, 2004; Kanayama et al., 2009; Marotta et al., 2016), even to the extent that approximately one-third of participants fail to experience the illusion (e.g., Ehrsson et al., 2004; Durgin et al., 2007; Lloyd, 2007). No doubt to some degree this variability is explainable by multi-sensory processes, duly complemented by a normative model of the body and by integration with interoceptive processes. Ehrsson (2012, p. 783), however, acknowledges that currently we are unable to explain variability within or “immunity” to the illusion, and conjectures that these phenomena might be understood with respect to differences in the degree to which people rely on proprioceptive information. But multiple studies suggest that proprioception does not strongly correlate with the illusion (e.g., Zopf et al., 2010; Rohde et al., 2011) or, to that extent that the two do correlate, it is the illusion—the conscious experience—that causally affects proprioception, not the reverse (Abdulkarim and Ehrsson, 2016). This renders implausible the suggestion that greater reliance upon proprioception could play the conjectured role.

Of course some further tweaking of the MSH might enhance its explanatory adequacy, but the problem may reside elsewhere, perhaps with the disinclination to consider a possible role played by higher cognitive functions. Although the received view of multisensory integration is that it is automatic and pre-attentive, a variety of theoretical considerations and recent experimental findings suggest that top-down attention can interact with integrative processes so as to enhance clarity, reduce ambiguity, facilitate feature-binding, and so forth (Talsma et al., 2012). Indeed, an association between bimodal integration and attention has been demonstrated already by several studies (Spence and Driver, 1997; Gondan et al., 2011).

Especially noteworthy are investigations of audiovisual integrative processing: Talsma et al. (2007) discovered that full attention to both modalities is necessary for successful integration. And, not only has the Talsma et al. finding been reconfirmed, attention has also been shown to play a role in modulating the activation of bimodal and unimodal regions—e.g., the superior temporal sulcus and the extrastriate cortex (Morís Fernández et al., 2015). In contrast, with the exception of Wahn and König (2015), far less scrutiny has been given to how attention or other higher cognitive functions might affect integration of touch and vision, to say nothing of proprioception. Accordingly, in view of the explanatory failings encountered when higher cognitive functions are neglected as well as findings concerning the influence of top-down attention, we determined to investigate the possibility that executive functions are modulating the RHI.

Among various executive functions, attention-related constructs like mind wandering and task switching appear to be especially relevant to the RHI. Mind wandering occurs when executive components of attention shift away from external tasks to internal thoughts and feelings (Smallwood and Schooler, 2006, 2015). It signals a failure of executive control to block the interference of those internal thoughts on a primary external task (McVay and Kane, 2010) and, at the same time, it signals a redirection of those executive resources to endogenously generated mental contents (Christoff et al., 2009). Task switching, on the other hand, which is taken to be at the very “nexus” of executive control (Schneider and Logan, 2009), enables people to redirect their attention away from the endogenous and back to the external (Hofmann et al., 2012).

Conceptually, there might be some resistance to treating the RHI as involving a task, especially since the focus here is only on experienced ownership of a body part, not an action, which are distinct types of belonging experience (Tsakiris et al., 2010a,b; Lane, 2014). But task switching can be thought of as an indicator of cognitive flexibility (Diamond, 2013), and on some accounts the RHI self-reported questionnaires are taken to be measurements more of judged rather than experienced ownership (de Vignemont, 2011b). If we allow that judgment plays some role in the RHI, then we have opened the door to higher cognitive functions, and flexibility of function is an important part of enabling a subject to report that a rubber hand belongs to self.

Yet, to our knowledge, no study has examined directly the degree to which these executive functions might influence multisensory integration. Hence, the present study is designed to do just that, to investigate the relationship among the RHI, task switch, and mind wandering. We conjecture that a role of executive functions in the RHI is to refocus participants' attention to external task-related stimuli, resulting in stronger RHI experiences. On the other hand, we conjecture that it is less likely for mind wanderers to experience the rubber hand belonging to self. We further conjecture that people who are better at task switching might be more efficient at refocusing attention onto the illusory experience, which may in turn enhance the vividness and quicken the onset time of the RHI.

To complement task switch and mind wandering experiments, because top-down attention plays a significant role in audiovisual integration, we added an Attentional Control Scale, in order to determine whether persons who are better at attentional control would also have a more pronounced RHI experience. Attentional Control Scale items comprise two parts: attention-focus and attention-shift. As the terms suggest, attention-focus items are used to estimate participants' capacity to stay engaged, and attention-shift items are used to estimate participants' aptitude for shifting attention. Based on our conjecture that higher executive function enables individuals to refocus their attention to the external task-related stimuli—stroking of the rubber and biological hands—we expected that participants with better attentional control would also evince early onset and robust experience of the RHI.

It should be born in mind that the purpose of our investigation is to provide a sufficient explanation of a particular type of conscious experience—the sensation that a rubber hand belongs to self. The role that we suggest is being played by top-down attentional mechanisms is consistent with the role played by top-down attention in more comprehensive theories of conscious experience, such as the global neuronal workspace hypothesis (e.g., Dehaene et al., 2006; Tsakiris et al., 2010b): that is, top-down attention seems to be a necessary condition that enables preconscious or subliminal processes to become consciously reportable. Although we are agnostic as to whether this hypothesis succeeds as a general theory of consciousness, it does seem applicable to the RHI.

Our hypothesis is that the role of executive function in RHI is to refocus cognitive resources from internal thoughts to external, task-related stimuli. Participants with higher mind wandering rates should respond to the RHI more slowly and experience it less robustly, as a result of their inclination to attend to internal thoughts rather than the external stimuli. In shifting attention to the external stimuli, there should be switch costs that accompany the shift. By hypothesis, participants with less switch cost are expected to respond to RHI faster. Moreover, the strength of the RHI correlates with whether participants are able to shift attention to external stimuli.

## Methods

### Participants

Thirty-six adults, including students and members of the general public, were recruited from the National Taiwan University and surrounding neighborhoods. All gave informed consent, had normal or suitably corrected-vision, were compensated for their participation, and were naïve to the purpose of RHI experiment. The study followed Human Subject Ethics Guidelines and was approved by the Ethics Committee of the Department of Psychology at the National Taiwan University.

### Stimuli and apparatus

#### Rubber hand illusion

All participants were tested individually in a small and quiet room. Since earlier work suggests a right hemispheric dominance for body ownership experience (Ocklenburg et al., 2011), our current experiment was administered only to each participant's left hand and a corresponding rubber hand was employed. The experimenter stood in front of each seated participant who was asked to extend both arms on the table with palms facing down. Then, each participant inserted his/her actual left hand into a black cardboard tube so the left hand would be out of sight, from the participant's perspective. The rubber hand was placed next to the hidden real hand, within the participant's field of vision. A blanket was placed over the tube such that it covered each participant's elbow and forearm, as well as the space separating the rubber hand from his/her torso. Finally, the experimenter used two paintbrushes to stroke both the actual and the rubber hand.

Each participant was exposed to two conditions with order counterbalanced: synchronous and asynchronous. In both conditions, the experimenter stroked the participant's real hand and the rubber hand from thumb to little finger and back, at a pace of approximately once per 2 s, for 3 min. In the synchronous condition, the experimenter stroked both the real hand and the rubber hand simultaneously. In the asynchronous condition, the experimenter alternated between the real hand and the rubber hand. Botvinick and Cohen (1998) found that participants reported an experience of ownership for the rubber hand when strokes were applied in synchrony. Because the ownership experience does not occur when stokes are applied in an asynchronous manner, it seems that synchronization of visual and tactile information is essential for the RHI (cf. Constantini et al., 2016). Therefore, we follow the convention in the RHI literature to use the asynchronous condition as the baseline to ensure that the RHI is indeed induced by the synchronous strokes, and not by visual or tactile sensations alone (or both). Two foot-pedals placed under the participant's feet were connected to a computer in order to measure the onset time of subjective ownership or belonging, which is the specific aspect of the RHI that we investigated. Participants were instructed to step on the left pedal as soon as the stroking began and to step on the right pedal as soon as they began to experience the rubber hand as part of self.

Touch referral is, arguably, the most distinctive perceptual event associated with the illusion, but the RHI can be induced even when participants cannot see the hand, and the experience of ownership has a richer phenomenology (Ehrsson, 2012). Accordingly, we concerned ourselves with ownership, the respect in which the rubber hand is experienced as belonging to self (Lane, 2012, 2015). Unfortunately, several studies have raised doubts about the reliability of proprioceptive drift, a commonly used behavioral proxy, as an indicator of the ownership experience for the rubber hand (e.g., Ehrsson et al., 2004; Kammers et al., 2009; Zopf et al., 2010; Rohde et al., 2011). Indeed, it even seems to be the case that when drift and ownership experience are positively correlated, it is the conscious experience that causes drift, not the reverse (Abdulkarim and Ehrsson, 2016).

Accordingly, for the purpose of investigating the ownership experience, we developed a new, online proxy. We discovered that asking participants to indicate the moment they begin to experience the rubber hand as belonging to self is a highly reliable measure of illusion strength (Lane et al., 2017). This technique enabled us to investigate the RHI's temporal dynamics, providing in-the-moment rather than only retrospective data, thereby introducing a new methodological approach.

In fact, despite the highly suggestive environment of the RHI induction, many participants, nevertheless, reported no sensation whatsoever, showing that this technique is a useful tool for measuring variation with the RHI. Moreover, we have previously established that onset time can be effectively employed in concert with questionnaires (Lane et al., 2017), such as the nine-item questionnaire created by Botvinick and Cohen (1998). As indicated by Botvinick and Cohen (1998), only items one to three exhibited statistical significance in production of affirmative responses. Therefore, for this study we measured the strength or intensity of the RHI (Morgan et al., 2011) by only taking the mean of items one to three in the Botvinick and Cohen questionnaire. Also, just as in Botvinick and Cohen (1998), each item is rated from −3 to 3.

#### Mind wandering

In order to determine individual variation in mind wandering—the degree to which minds wander from the current task to private thoughts or feelings (Smallwood and Schooler, 2006)—we used a modified version of the Robertson et al. (1997) Sustained Attention to Response Task (SART). It is a continuous “Go” or “No-go” task that requires participants to respond to all non-targets (letters of the alphabet, except C) by pressing down on the space bar as quickly as possible. When the target, the letter “C,” appears, participants were required to inhibit response (viz., perform no action). The target to non-target ratio was 1:19. During the administration of SART, 30 thought probes were inserted pseudo-randomly. These probes comprised three options, all pertaining to what participants were thinking about immediately prior to the probe. Participants were required to indicate whether their thoughts concerned (a) the task, (b) task performance, or (c) matters irrelevant to the task. Thus, mind wandering is defined as the degree to which participants' thoughts prior to the probe are irrelevant to the task, and the degree of mind wandering was calculated as the ratio of task-irrelevant responses for the 30 thought probes.

#### Task switching

**Figure 1:**
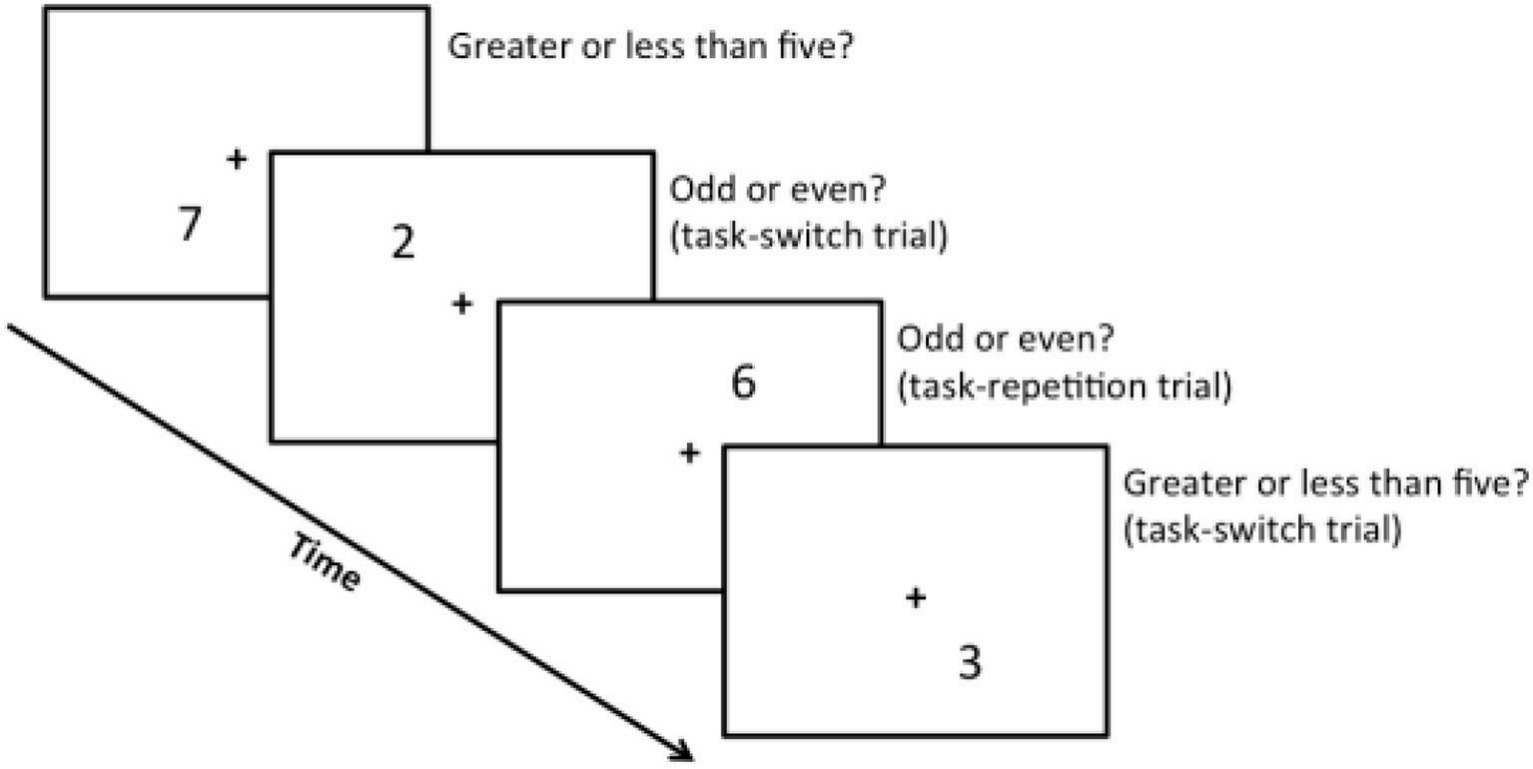
An example of the task switch procedure. A fixation cross is situated at the center of a screen and a series of digits (1–4, 6–9) appear in the surrounding quadrants. Participants were asked to press key “A” on a standard qwerty keyboard when digits were odd and on the upper field, or when digits were greater than five and on the lower field; participants were asked to press key “;” on the same standard qwerty keyboard when digits were even and on the upper field, or less than five and on the lower field. Consecutive tasks that require the same type of judgment—e.g., deciding whether upper field digits are odd–count as task-repetition trials. Consecutive tasks that require different types of judgment—e.g., first deciding whether a lower field digit is greater than five, then deciding whether an upper field digit is odd–count as task-switch trials.

Similar to the standard task switch paradigm, participants were asked to make binary judgments. They had to decide (1) whether digits that appeared on the screen were odd or even (when they appeared on the upper field), or (2) whether they were greater than or less than five (when they appeared on the lower field). Ability to switch between the two tasks was assessed by comparing the reaction times (RTs) in task-switch trials (switching from one task to another) and task-repetition trials (consecutive performance of the same task). See Figure 1 for details (adapted from Erickson et al., 2008). We predicted that task-switch trials would prolong RT relative to task-repetition trials. Thus, adeptness at switching could be determined by calculating the average RT delay for all participants—individuals with shorter delay would be more proficient at switching.

Participants were first given one practice block of 30 trials. Next, we gave them a total of four blocks, each of which comprised 60 trials. We then measured the RTs of the task-repetition and task-switch trials, and calculated the switch cost (i.e., the difference between mean RTs of task-switch trials and task-repetition trials).

#### Attentional control scale

The Attentional Control Scale (see Appendix) is designed to explore distinct aspects of attentional control (Derryberry and Reed, 2002): items one to nine concern the ability to attention-focus; items 10–20 concern the ability to attention-shift. Attention-focus items are subjective reports of a participant's capacity to stay engaged (e.g., “When trying to focus my attention on something, I have difficulty blocking out distracting thoughts”). On the other hand, attention-shift items are subjective reports of a participant's aptitude for shifting attention from one task to another (e.g., “It takes me a while to get really involved in a new task”). After completing the experiment, two scores were calculated: the average for attention-focus and the average for attention-shift.

### Design and procedure

Administration order of the RHI, Mind Wandering, and Task Switch were counterbalanced across participants. The Attentional Control Scale was administered after the first three had been completed.

## Results

### Rubber hand illusion

**Figure 2:**
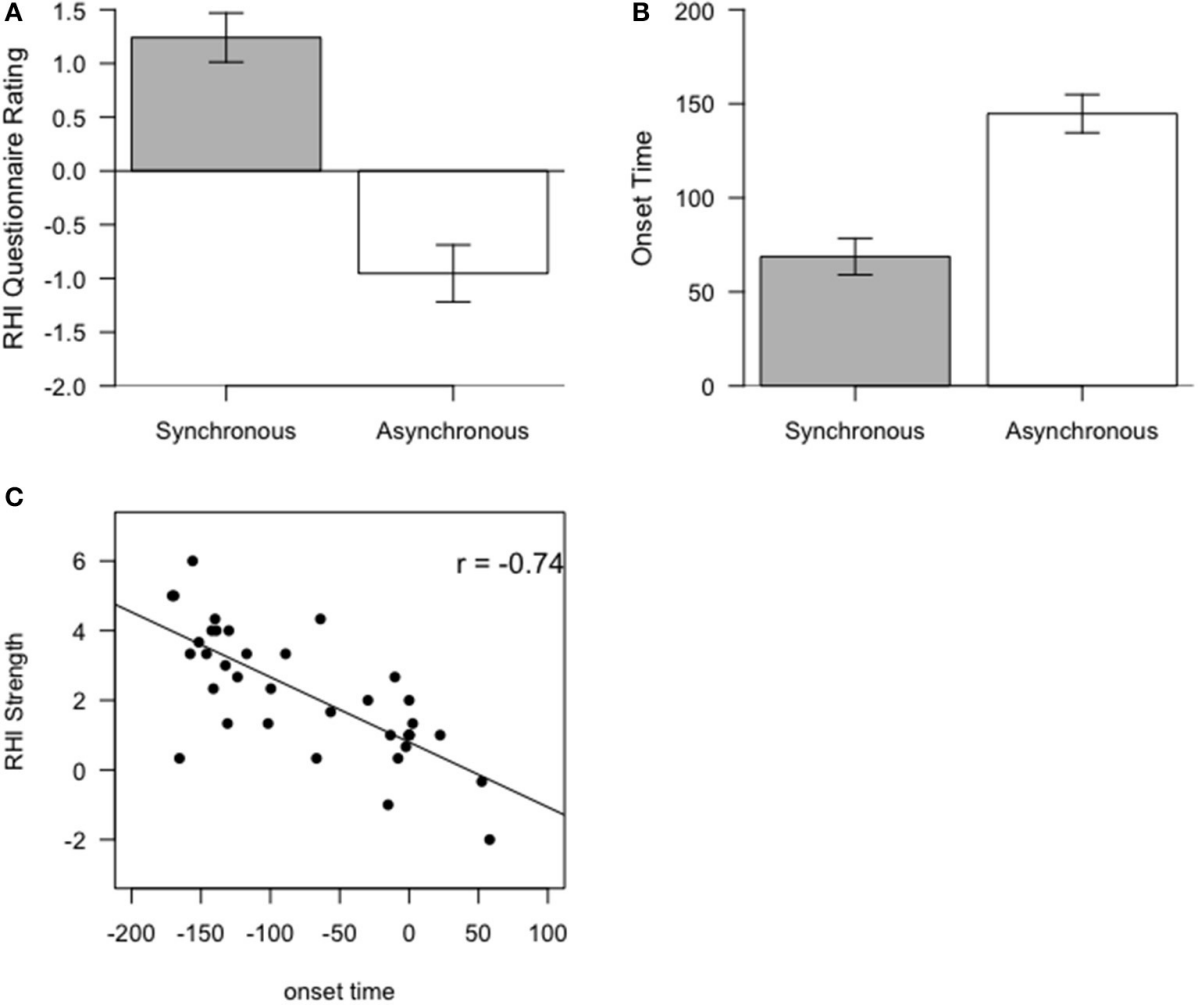
**(A)** The difference in RHI questionnaire ratings between synchronous and asynchronous conditions **(B)** The difference in onset time between synchronous and asynchronous conditions. The error bars in both **(A)** and **(B)** represent standard errors (SE) of the mean. **(C)** Correlation between RHI strength—the difference in mean for items one to three, as rated by RHI questionnaire between synchronous and asynchronous conditions—and onset time for RHI.

To analyze the RHI questionnaire ratings, a *t*-test was performed in order to test the difference between the mean of items one to three under the synchronous and the asynchronous conditions (Figure 2A). The main effect, reflecting this difference, was significant [*t*_(35)_ = 7.3944, *p* < 0.001]. This finding suggests that participant's RHI experience was stronger or more intense when stroking was synchronous than when it was asynchronous. For the onset time data, the *t*-test that concerned the difference between synchronous and asynchronous stroking (Figure 2B) showed that the main effect, reflecting this difference, was significant [*t*_(35)_ = −6.3785, *p* < 0.001]. This result suggests that participants experienced the illusion (viz., feeling the rubber hand belongs to self) quicker when stroking was synchronous than when it was asynchronous.

Additionally, we discovered a correlation between mean RHI, as indicated by onset time for ownership, and RHI strength, as indicated by the mean of items one to three (*r* = −0.74, *p* < 0.001, Figure 2C), when the asynchronous condition was subtracted from the synchronous condition. The RHI strength was calculated in this manner because we took the score in the asynchronous conditions as the baseline. This correlation implies that the more vivid or the stronger the experience of the RHI, the earlier is the onset of experienced ownership for the rubber hand. These findings concerning the relationship between questionnaire and onset time ratings are consistent with our previous findings (Lane et al., 2017).

### Hierarchical regression analyses

To investigate how the RHI experience was affected by different aspects of executive function, we conducted two hierarchical regressions with the onset time of the RHI and the RHI questionnaire ratings as dependent variables respectively. The regression analysis of the RHI onset time could reveal how executive functions affect the temporal dynamics of experiencing the RHI, whereas the regression analysis of the RHI questionnaire ratings could reveal how executive functions affect the intensity of the RHI.

Based on the hypothesis mentioned in the Introduction section, we entered executive function predictors in the following order: (1) the SART score in the mind wandering task, (2) the attention-shift rating in the attentional control scale, (3) the switch cost in the task switching task, and (4) the attention-focus rating in the attentional control scale.

### Hierarchical regression of the onset time of RHI

**Figure 3:**
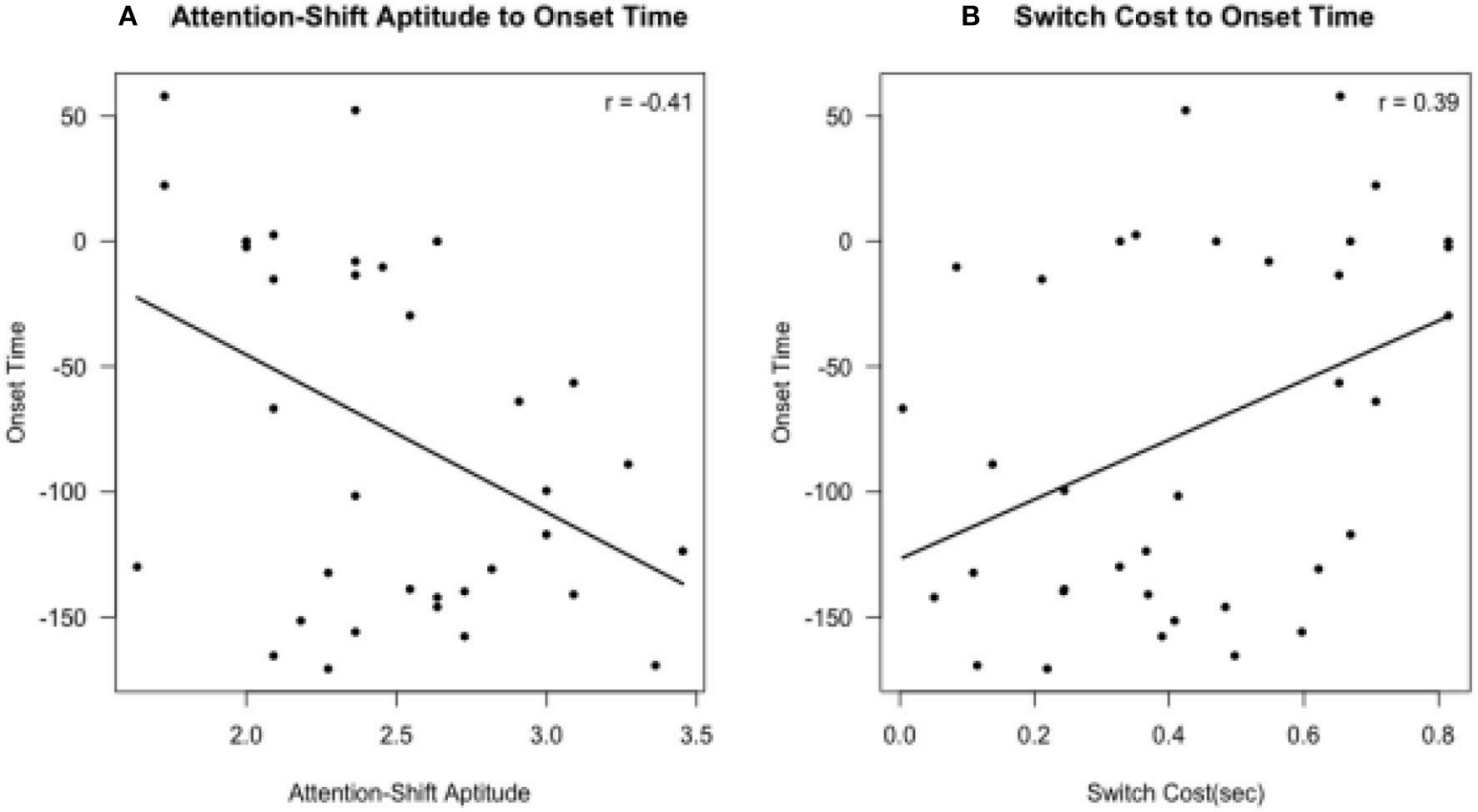
Correlations between the RHI onset time and **(A)** Attention-Shift Aptitude **(B)** Switch Cost (s) in Model 3.

The results of the hierarchical regression on the RHI onset time are shown in Table 1. These results showed that the increased *R*^2^ between Model 2 and Model 1 [*F*_(1, 33)_ = 6.7606, *p* < 0.05], and the increased *R*^2^ between Model 3 and Model 2 [*F*_(1, 32)_ = 4.7496, *p* < 0.05] were both significant, but the increased *R*^2^ between Model 3 and Model 4 was not significant [*F*_(1, 31)_ = 0.8113, *p* = 0.3747]. That is, Model 3 explains the onset time of the RHI better than Model 2 and Model 1; however, Model 4 does not explain the onset time better than Model 3. Therefore, we chose Model 3 to explain the results of the RHI onset time, since Model 3 could explain the variance of the onset time with fewer predictors. In Model 3, the attention-shift ratings were negatively related to the onset time of the RHI [*t*_(32)_ = −2.243, *p* < 0.05], while the switch costs were positively related to the onset time of the RHI [*t*_(32)_ = 2.186, *p* < 0.05]. This suggests that participants who could shift their attention from one task to another with less switch cost could respond more quickly to the RHI experience (Figure 3).

**Table 1:** Results of the hierarchical regression analysis of the RHI onset time.

**Predictor**	**Model 1**	**Model 2**	**Model 3**	**Model 4**
Mind wandering (SART score)	31.17	−8.926	−7.172	−8.925
Attention-shift rating		−64.849^*^	−56.449^*^	−45.156^*^
Task switch (switch cost)			101.699^*^	106.271
Attention-focus rating				−28.467
*R^2^*	0.0145	0.1683	0.2764	0.2948
*ΔR^2^*		0.1538	0.1081	0.0184
*F*	0.502	3.34^*^	4.074^*^	3.24^*^
*F of increased R^2^*		6.7606^*^	4.7496^*^	0.8113

* *p < 0.05*.

### Hierarchical regression of the RHI questionnaire ratings

**Figure 4:**
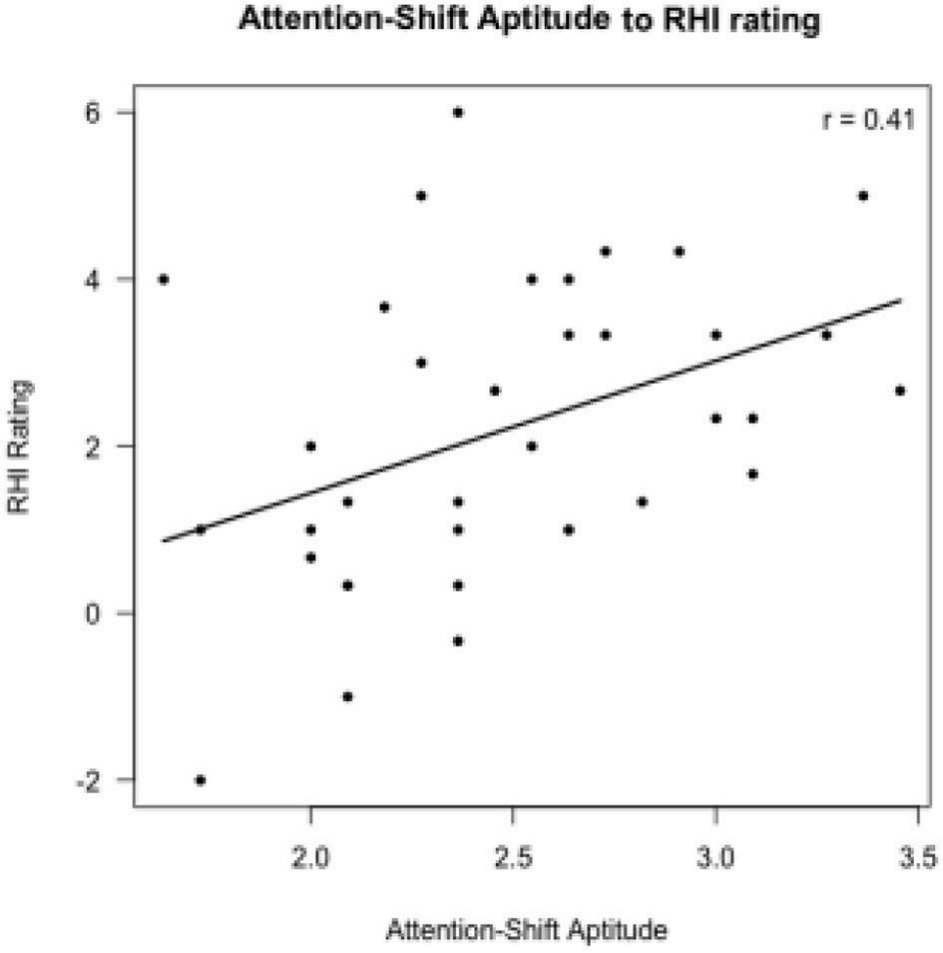
Correlations between the RHI questionnaire rating and attention-shift aptitude in Model 2.

The results of the hierarchical regression of the RHI questionnaire ratings are shown in Table 2. These results showed that only the difference of *R*^2^ between Model 1 and Model 2 was significant [*F*_(1, 33)_ = 3.346, *p* < 0.05]. This finding suggests that Model 2 explains the variance of RHI questionnaire ratings better than Model 1. However, Model 3 and 4 do not explain the RHI questionnaire ratings better than Model 2. In Model 2, the attention-shift ratings were positively related to the RHI questionnaire ratings [*t*_(31)_ = 2.510, *p* < 0.05], suggesting that the participants with higher attention-shift ratings have stronger RHI experience (Figure 4).

**Table 2:** Results of hierarchical regression analysis of The RHI questionnaire rating.

**Predictor**	**Model 1**	**Model 2**	**Model 3**	**Model 4**
Mind wandering (SART score)	−0.6511	0.3788	0.3499	0.4056
Attention-shift rating		1.6658^*^	1.5274	1.1686
Task switch (switch cost)			−1.6758	−1.8210
Attention-focus rating				0.9044
*R^2^*	0.0099	0.1686	0.2145	0.2436
*ΔR^2^*		0.1587	0.0459	0.0291
*F*	0.341	3.346^*^	2.912	2.496
*F of increased R^2^*		6.5029^*^	1.8800	1.1937

* *p < 0.05*.

In short, the results suggest that participants who are more adept at shifting attention to the new task with less switch cost would respond to the RHI faster (as indicated by the RHI onset time), and the participants with higher attention-shift ratings would experience stronger RHI (as indicated by the questionnaire ratings). Together, our results suggest that cognitive flexibility is a key factor for the RHI. The ability of shifting attention toward external visual-tactile stimulation affects both the RHI onset time and strength.

## General discussion

Previously it has been supposed that MSH, when appropriately supplemented, could provide sufficient explanations for the RHI (Ehrsson, 2012). Partially in the interest of parsimony, and partially because the additions made to MSH (e.g., Tsakiris, 2011, 2017; Moseley et al., 2012) seemed up to the task of increasing MSH's explanatory adequacy and scope, higher cognitive functions were neglected. The intent of our investigations has not been to suggest that multisensory integration is inessential to experience of the RHI, nor indeed to suggest that a pre-existing body model or interoception do not contribute importantly to solving the explanatory puzzles of embodiment. Instead, our intent has been to suggest that an adequate explanation of the variable experiences of those who undergo the RHI induction procedure will need to take into account another factor—executive and other higher cognitive functions.

Discussion of executive function's role in this respect may be counter-intuitive, since all healthy participants know that a detached rubber hand could not belong to self. One might even be inclined to think there is a need to inhibit executive functions in order to experience the RHI, given the patent absurdity of bodies adopting disconnected parts. But our findings suggest that adequate explanation of the presence of the illusion intensity variation that is so often reported (e.g., Durgin et al., 2007; Kanayama et al., 2009) might require inclusion of executive functions playing specific roles. Foremost among these are attention-involving executive functions, including task switch and attention shift. Both seem to be playing a modulatory role. Indeed, it might be that a certain degree of the cognitive flexibility that these contribute to our cognitive economy is a prerequisite for the having of such an illusory experience.

Filippetti and Tsakiris (2017) recently showed that participants' accuracy on reporting their heartbeats, a performance index of the ability to perceive internal bodily signals, was improved *after* experiencing the RHI. Improvement of interoceptive awareness, however, was only observed for participants who exhibited relatively low accuracy *before* the RHI experience. The authors suggest that body-ownership is a hierarchical construct that is dependent upon multisensory signals, and that synchronous visual-tactile stimulation can improve the performance of participants on tasks of interoceptive awareness. Their finding is consistent with previous findings that attention to exteroceptive bodily signals enhances processing of self-related information (Tsakiris et al., 2007, 2011; Ainley et al., 2012; Maister and Tsakiris, 2014). These findings seem to be compatible with ours, in the following way: if task switch and attention shift are understood as indicators of cognitive flexibility that are trainable, which appears to be the case (Karbach and Kray, 2009; Diamond, 2013), then the improvement of interoceptive awareness might be best understood as a result of the RHI “training” sessions, sessions that promote the ability to attend, flexibly, to sensory signals.

In this study, participants always filled in the Attentional Control Scale after they completed all RHI, mind wandering, and task switch experiments. One might wonder whether this sequence biased participants' responses. But items in the Attentional Control Scale are not directly related to those tasks; instead, they measure one's attention capability, in general. Therefore the observed effects in the present study should not be influenced by the fixed order of the tasks and questionnaire.

One of the limitations of this study is that it concerns body image representations, and is silent as regards body schema representations. The standard RHI protocol, the one employed here, comprises passive tactile stimulation and subjective report; hence, it seems only to inform about body image, not body schema, which typically involves sensorimotor coordination (de Vignemont, 2010). In principle image and schema can dissociate, but since our experiment involved no reaching, grasping, or pointing, we leave for future studies to determine whether executive functions can similarly modulate body schema representations.

A second, further line of investigation suggested by our findings concerns cognitive flexibility. In our effort to explain variability of illusion experience, we have suggested that higher cognitive functions are playing a modulatory role and that a degree of cognitive flexibility might be a prerequisite for the having of illusory conscious experiences of the sort on display in the RHI. Because task switch and attention shift seem to be trainable, we conjecture that relevant training, by promoting the ability to attend, flexibly, to sensory signals, should enhance performance on the RHI.

In the history of science, parsimony is widely regarded as a pragmatic virtue, sometimes even as an epistemic virtue (Quine, 1966). But premature ambitions to parsimony can inhibit the development of science, resulting in theories of limited explanatory scope. By taking higher cognitive functions into consideration and by recognizing that executive functions play an important role in the RHI, we position ourselves to better explain variation in the experience of ownership, perhaps even better explain why it is that one-third of people who undergo the RHI induction fail to experience any illusion whatsoever.

## Author contributions

S-LY and TL conceived the idea and designed this study. A-YC and S-EC conducted the experiment and analyzed the data. S-LY, TL, and S-EC wrote the manuscript.

### Conflict of interest statement

The authors declare that the research was conducted in the absence of any commercial or financial relationships that could be construed as a potential conflict of interest.

## Appendix

Attentional Control Scale. Items 1, 2, 3, 6, 7, 8, 11, 12, 15, 16, and 20 were coded inversely, consistent with the method employed by Derryberry and Reed (2002).

**Table:**

Focus attention	1.	It's very hard for me to concentrate on a difficult task when there are noises around.
	2.	When I need to concentrate and solve a problem, I have trouble focusing my attention
	3.	When I am working hard on something, I still get distracted by events around me.
	4.	My concentration is good even if there is music in the room around me.
	5.	When concentrating, I can focus my attention so that I become unaware of what's going on in the room around me.
	6.	When I am reading or studying, I am easily distracted if there are people talking in the same room
	7.	When trying to focus my attention on something, I have difficulty blocking out distracting thoughts.
	8.	I have a hard time concentrating when I'm excited about something.
	9.	When concentrating, I ignore feelings of hunger or thirst.
Shift attention	10.	I can quickly switch from one task to another.
	11.	It takes me a while to get really involved in a new task.
	12.	It is difficult for me to coordinate my attention between listening and writing required when taking notes during lectures.
	13.	I can become interested in a new topic very quickly when I need to.
	14.	It is easy for me to read or write while I'm also talking on the phone.
	15.	I have trouble carrying on two conversations at once.
	16.	I have a hard time coming up with new ideas quickly.
	17.	After being interrupted or distracted, I can easily shift my attention back to what I was doing before.
	18.	When a distracting thought comes to mind, it is easy for me to shift my attention away from it.
	19.	It is easy for me to alternate between two different tasks.
	20.	It is hard for me to break from one way of thinking about something and look at it from another point of view.

## References

[B1] Abdulkarim Z. Ehrsson H. H. (2016). No causal link between changes in hand position sense and feeling of limb ownership in the rubber hand illusion. Attent. Percept. Psychophys. 78, 707–720. 10.3758/s13414-015-1016-0 26555651 PMC4744264

[B2] Ainley V. Tajadura-Jiménez A. Fotopoulou A. Tsakiris M. (2012). Looking into myself: changes in interoceptive sensitivity during mirror self-observation. Psychophysiology 9, 1672–1676. 10.1111/j.1469-8986.2012.01468.x PMC3755258 22978299

[B3] Armel K. C. Ramachandran V. S. (2003). Projecting sensations to external objects: evidence from skin conductance response. Proc. R. Soc. Lond. Biol. Sci. 270, 1499–1506. 10.1098/rspb.2003.2364 12965016 PMC1691405

[B4] Barnsley N. McAuley J. H. Mohan R. Dey A. Thomas P. Moseley G. I. (2011). The rubber hand illusion increases histamine reactivity in the real arm. Curr. Biol. 21, R945–R946. 10.1016/j.cub.2011.10.039 22153159

[B5] Botvinick M. (2004). Probing the neural basis of body ownership. Science 305, 782–783. 10.1126/science.1101836 15297651

[B6] Botvinick M. Cohen J. (1998). Rubber hands 'feel' touch that eyes see. Nature 391:756. 10.1038/35784 9486643

[B7] Christoff K. Gordon A. M. Smallwood J. Smith R. Schooler J. W. (2009). Experience sampling during fMRI reveals default network and executive system contributions to mind wandering. Proc. Natl. Acad. Sci. U.S.A. 106, 8719–8724. 10.1073/pnas.0900234106 19433790 PMC2689035

[B8] Constantini M. Haggard P. (2007). The rubber hand illusion: sensitivity and reference frame for body ownership. Conscious. Cogn. 16, 229–240. 10.1016/j.concog.2007.01.001 17317221

[B9] Constantini M. Robinson J. Migliorati D. Donno B. Ferri F. Northoff G. (2016). Temporal limits on rubber hand illusion reflect individuals' temporal resolution in multisensory perception. Cognition 157, 39–48. 10.1016/j.cognition.2016.08.010 27592410

[B10] de Vignemont F. (2010). Body schema and body image-Pros and cons. Neuropsychologia 48, 669–680. 10.1016/j.neuropsychologia.2009.09.022 19786038

[B11] de Vignemont F. (2011a). A self for the body. Metaphilosophy 42, 230–247. 10.1111/j.1467-9973.2011.01688.x

[B12] de Vignemont F. (2011b). Embodiment, ownership, and disownership. Conscious. Cogn. 20, 81–93. 10.1016/j.concog.2010.09.004 20943417

[B13] Dehaene S. Changeux J. P. Naccache L. Sackur J. Sergent C. (2006). Conscious, preconscious, and subliminal processing: a testable taxonomy. Trends Cogn. Sci. 10, 204–211. 10.1016/j.tics.2006.03.007 16603406

[B14] Derryberry D. Reed M. A. (2002). Anxiety-related attentional biases and their regulation by attentional control. J. Abnorm. Psychol. 111, 225–236. 10.1037/0021-843X.111.2.225 12003445

[B15] Diamond A. (2013). Executive functions. Annu. Rev. Psychol. 64, 135–168. 10.1146/annurev-psych-113011-143750 23020641 PMC4084861

[B16] Durgin F. H. Evans L. Dunphy N. Klostermann S. Simmons K. (2007). Rubber hands feel the touch of light. Psychol. Sci. 18, 152–157. 10.1111/j.1467-9280.2007.01865.x 17425536

[B17] Ehrsson H. H. (2012). The concept of body ownership and its relation to multisensory integration, in The New Handbook on Multisensory Processing, ed Stein B. E. (Cambridge, MA: The MIT Press), 775–793.

[B18] Ehrsson H. H. Spence C. Passingham R. E. (2004). That's my hand! Activity in premotor cortex reflects feeling of ownership of a limb. Science 305, 875–877. 10.1126/science.1097011 15232072

[B19] Erickson K. I. Kim J. S. Suever B. L. Voss M. W. Francis B. M. Kramer A. F. (2008). Genetic contributions to age-related decline in executive function: a 10-year longitudinal study of COMT and BDNF polymorphisms. Front. Hum. Neurosci. 2:11. 10.3389/neuro.09.011.2008 18958211 PMC2572207

[B20] Filippetti M. L. Tsakiris M. (2017). Heartfelt embodiment: changes in body-ownership and self-identification produce distinct changes in interoceptive accuracy. Cognition 159, 1–10. 10.1016/j.cognition.2016.11.002 27880880

[B21] Gondan M. Blurton S. P. Hughes F. Greenlee M. W. (2011). Effects of spatial and selective attention on basic multisensory integration. J. Exp. Psychol. Hum. Percept. Perform. 37, 1887–1897. 10.1037/a0025635 21967270

[B22] Grivaz P. Blanke O. Serino A. (2017). Common and distinct brain regions processing multisensory bodily signals for peripersonal space and body ownership. Neuroimage 147, 602–618. 10.1016/j.neuroimage.2016.12.052 28017920

[B23] Hofmann W. Schmeichel B. J. Baddeley A. D. (2012). Executive functions and self-regulation. Trends Cogn. Sci. 16, 174–180. 10.1016/j.tics.2012.01.006 22336729

[B24] Kammers M. P. Verhagen L. Dijkerman H. C. Hogendoorn H. de Vignemont F. Schutter D. J. (2009). Is this hand for real? Attenuation of the rubber hand illusion by transcranial magnetic stimulation over the inferior parietal lobe. J. Cogn. Neurosci. 21, 1311–1320. 10.1162/jocn.2009.21095 18752397

[B25] Kanayama N. Sato A. Ohira H. (2009). The role of gamma band oscillations and synchrony on rubber hand illusion and crossmodal integration. Brain Cogn. 69, 19–29. 10.1016/j.bandc.2008.05.001 18555572

[B26] Karbach J. Kray J. (2009). How useful is executive control training? Age differences in near and far transfer of task-switching. Dev. Sci. 12, 978–990. 10.1111/j.1467-7687.2009.00846.x 19840052

[B27] Lane T. (2012). Toward an explanatory framework for mental ownership. Phenomenol. Cogn. Sci. 11, 251–286. 10.1007/s11097-012-9252-4

[B28] Lane T. (2014). When actions feel alien: an explanatory model, in Communicative Action, ed Hung T. W. (Singapore: Springer Science+Business Media), 53–74.

[B29] Lane T. (2015). Self, belonging, and conscious experience: a critique of subjectivity theories of consciousness, in Disturbed Consciousness: New Essays on Psychopathology and Theories of Consciousness, ed Gennaro R. (Cambridge, MA: MIT Press), 103–140.

[B30] Lane T. Yeh S.-Y. Tseng P. Chang A.-Y. (2017). Timing disownership experiences in the rubber hand illusion. Cogn. Res. Princ. Impl. 20172:4 10.1186/s41235-016-0041-4 PMC5281674 28203632

[B31] Lipton P. (2004). Inference to the Best Explanation, 2nd Edn. London: Routledge.

[B32] Lloyd D. M. (2007). Spatial limits on referred touch to an alien limb may reflect boundaries of visuo-tactile peripersonal space surrounding the hand. Brain Cogn. 64, 104–109. 10.1016/j.bandc.2006.09.013 17118503

[B33] Maister L. Tsakiris M. (2014). My face, my heart: cultural differences in integrated bodily self-awareness. Cogn. Neurosci. 5, 10–16. 10.1080/17588928.2013.808613 24168204

[B34] Makin T. R. Holmes N. P. Ehrsson H. H. (2008). On the other hand: dummy hands and peripersonal space. Behav. Brain Res. 191, 1–10. 10.1016/j.bbr.2008.02.041 18423906

[B35] Marotta A. Tinazzi M. Cavedini C. Zampini M. Fiorio M. (2016). Individual differences in the rubber hand illusion are related to sensory suggestibility. PLoS ONE 11:e0168489. 10.1371/journal.pone.0168489 27977783 PMC5158054

[B36] McVay J. C. Kane M. J. (2010). Does mind wandering reflect executive function or executive failure? Comment on Smallwood and Schooler (2006) and Watkins (2008). Psychol. Bull. 136, 188–197. 10.1037/a0018298 20192557 PMC2850105

[B37] Melzack R. (1992). Phantom limbs. Sci. Am. 266, 120–126. 10.1038/scientificamerican0492-120 1566028

[B38] Morgan H. L. Turner D. C. Corlett P. R. Absalom A. R. Adapa R. Arana F. S.. (2011). Exploring the impact of ketamine on the experience of illusory body ownership. Biol. Psychiatry 69, 35–41. 10.1016/j.biopsych.2010.07.032 20947068 PMC3025328

[B39] Morís Fernández L. Visser M. Ventura-Campos N. Ávila C. Soto-Faraco S. (2015). Top-down attention regulates the neural expression of audiovisual integration. Neuroimage 119, 272–285. 10.1016/j.neuroimage.2015.06.052 26119022

[B40] Moseley G. L. Gallace A. Spence C. (2012). Bodily illusions in health and disease: physiological and clinical perspectives and the concept of a cortical ‘body matrix’. Neurosci. Biobehav. Rev. 36, 34–46. 10.1016/j.neubiorev.2011.03.013 21477616

[B41] Ocklenburg S. Ruther N. Peterburs J. Pinnow M. Gunturkun O. (2011). Laterality in the rubber hand illusion. Laterality 16, 174–187. 10.1080/13576500903483515 20198533

[B42] Quine W. V. O. (1966). On simple theories of a complex world, in The Ways of Paradox. New York, NY: Random House.

[B43] Robertson I. H. Manly T. Andrade J. Baddeley B. T. Yiend J. (1997). ‘Oops!’: performance correlates of everyday attentional failures in traumatic brain injured and normal subjects. Neuropsychologia 35, 747–758. 10.1016/S0028-3932(97)00015-8 9204482

[B44] Rohde M. Di Luca M. Ernst M. O. (2011). The rubber hand illusion: feeling of ownership and proprioceptive drift do not go hand in hand. PLoS ONE 6:e21659. 10.1371/journal.pone.0021659 21738756 PMC3125296

[B45] Schneider D. W. Logan G. D. (2009). Task switching, in Encyclopedia of Neuroscience, Vol. 9, ed Squire L. R. (Oxford: Academic Press), 869–874.

[B46] Smallwood J. Schooler J. W. (2006). The restless mind. Psychol. Bull. 132, 946–958. 10.1037/0033-2909.132.6.946 17073528

[B47] Smallwood J. Schooler J. W. (2015). The science of mind wandering: empirically navigating the stream of consciousness. Annu. Rev. Psychol. 66, 487–518. 10.1146/annurev-psych-010814-015331 25293689

[B48] Spence C. Driver J. (1997). Audiovisual links in exogenous covert spatial orienting. Percept. Psychophys. 59, 1–22. 10.3758/BF03206843 9038403

[B49] Talsma D. Doty T. J. Woldorff M. G. (2007). Selective attention and audiovisual integration: is attending to both modalities a prerequisite for early integration? Cereb. Cortex 17, 679–690. 10.1093/cercor/bhk016 16707740

[B50] Talsma D. Senkowski D. Soto-Faraco S. Woldorff M. G. (2012). Influences of top-down attention on multisensory processing, in The New Handbook on Multisensory Processing, ed Stein B. E. (Cambridge, MA: The MIT Press), 371–382.

[B51] Tastevin J. (1937). En parent de L'experience D'aristotle. L'Encephale, I. 57–84.

[B52] Tsakiris M. (2010). My body in the brain: a neurocognitive model of body-ownership. Neuropsychologia 48, 703–712. 10.1016/j.neuropsychologia.2009.09.034 19819247

[B53] Tsakiris M. (2011). The sense of body ownership, in The Oxford Handbook of the Self, ed Gallagher Shaun (Oxford, UK: Oxford University Press), 180–203.

[B54] Tsakiris M. (2017). The multisensory basis of the self: from body to identity to others. Q. J. Exp. Psychol. 70, 4, 597–609. 10.1080/17470218.2016.1181768 PMC5214748 27100132

[B55] Tsakiris M. Carpenter L. James D. Fotopoulou A. (2010a). Hands only illusion: multisensory integration elicits sense of ownership for body parts but not for non-corporeal objects. Exp. Brain Res. 204, 343–352. 10.1007/s00221-009-2039-3 19820918

[B56] Tsakiris M. Constantini M. Haggard P. (2008). The role of the right temporoparietal junction in maintaining a coherent sense of one' body. Neuropsychologia 46, 3014–3018. 10.1016/j.neuropsychologia.2008.06.004 18601939

[B57] Tsakiris M. Hesse M. D. Boy C. Haggard P. Fink G. R. (2007). Neural signatures of body ownership: a sensory network for bodily self-consciousness. Cereb. Cortex 17, 2235–2244. 10.1093/cercor/bhl131 17138596

[B58] Tsakiris M. Longo M. R. Haggard P. (2010b). Having a body versus moving your body: neural signatures of agency and body-ownership. Neuropsychologia 48, 2740–2274. 10.1016/j.neuropsychologia.2010.05.021 20510255

[B59] Tsakiris M. Tajadura-Jiménez A. Constantini M. (2011). Just a heartbeat away from one's body: interoceptive sensitivity predicts malleability of body-representation. Proc. R. Soc. B Biol. Sci. 278, 2470–2476. 10.1098/rspb.2010.2547 PMC3125630 21208964

[B60] Wahn B. König P. (2015). Vision and haptics share spatial attentional resources and visuotactile integration is not affected by high attentional load. Multisens. Res. 28, 371–392. 10.1163/22134808-00002482 26288905

[B61] Zopf R. Savage G. Williams M. A. (2010). Crossmodal congruency measures lateral distance effects on the rubber hand illusion. Neuropsychologia 48, 713–725. 10.1016/j.neuropsychologia.2009.10.028 19913040

